# Importance of Social Support of Parents of Children with Cancer: A Multicomponent Model Using Partial Least Squares-Path Modelling

**DOI:** 10.3390/ijerph20031757

**Published:** 2023-01-18

**Authors:** Anabel Melguizo-Garín, María Dolores Benítez-Márquez, Isabel Hombrados-Mendieta, María José Martos-Méndez

**Affiliations:** 1 Department of Social Psychology, Social Work and Social Anthropology, University of Malaga, 29071 Málaga, Spain; 2 Department of Applied Economics, Statistics and Econometrics, University of Malaga, 29071 Málaga, Spain

**Keywords:** social support received, social support provided, adjustment of parents, stress, life satisfaction, parents of children with cancer, PLS-SEM, structural equation modelling

## Abstract

The purpose of the present study is to build a model combining some variables that have been previously studied separately to improve our understanding on how they relate in parents of children with cancer. A total of 112 parents with an average age of 41 completed the self-assessment questionnaires containing the factors studied: social support received, social support provided, stress, adjustment of parents and life satisfaction. Two models were developed: one for social support received and one for social support provided. Structural equation models based on the variance estimated through partial least squares were used to analyze factors involved in quality of life based on an exploratory model of second order. The estimated model was robust in terms of quality of measurement (reliability and validity). According to results from the structural model, in the model of social support received, the impact of social support received on stress was considerable (β = −0.26; *p* = 0.02) and it explained 16% of the variance. The impact of social support received by parents on their adjustment (β = −0.56; *p* < 0.001) was also considerable, explaining 32% of the variance. Finally, adjustment of parents also showed an effect on life satisfaction (β = −0.33; *p* < 0.001) and it explained 26% of the variance. However, the relation between social support received (β = 0.15; *p* = 0.11) and life satisfaction, the relation between stress (β = −0.15; *p* = 0.08) and life satisfaction, and the relation between adjustment of parents (β = 0.20; *p* = 0.07) and stress were not significant. In the model of social support provided by parents, social support provided (β = 0.35; *p* < 0.001), and adjustment of parents (β = −0.31; *p* < 0.01) impacted life satisfaction, explaining 36% of the variance. Social support provided (β = −0.34; *p* < 0.01) impacted adjustment of parents and it explained 12% of the variance. Adjustment of parents (β = 0.28; *p* < 0.05) also impacted parents’ perception of stress, explaining 14% of the variance. However, the relation between social support provided (β = −0.17; *p* = 0.06) and stress, and the relation between stress (β = −0.13; *p* = 0.08) and life satisfaction, were not significant. Social support received showed a strong connection with stress and parents’ adjustment. Additionally, social support received showed a decrease in stress and parents’ adjustment. Social support provided by parents and the adjustments they experience are linked to their life satisfaction. Additionally, social support provided showed a decrease in adjustment and an increase in parents’ life satisfaction. The models can be used to improve parents’ situations and it has strong practical implications.

## 1. Introduction

Childhood cancer implies deep changes in the social and family sphere [1] of the child with cancer and the parents. Parents of children with cancer face a series of circumstances that can lead to great changes in their lives [2,3] and which can generate stress in them [4].

Families who experience childhood cancer face a wide range of situations and challenges with a strong impact on their lives [5], such as frequent treatments and hospitalizations of the child, secondary effects resulting from treatments, uncertainty about the course of the cancer and fear of possible relapses [6,7]. These challenges increase parents’ stress levels [2,8] and, in fact, many parents often show symptoms linked to stress even after the child overcomes the cancer [4,9]. The main situations with the highest links to stress experienced by parents are those related to hospitalizations [10], receiving information about to the child [11] and waiting times for diagnoses and tests [12].

Childhood cancer alters the family system, and it affects parents’ quality of life [13,14], with some disruptions remaining over time [15]. The child with cancer depends on the care of one family member, who usually assumes the role of main carer [16]. Parents can experience family imbalances [17], particularly in what concerns the child with cancer and the siblings [18,19,20], and they can also see their social and economic-labor spheres altered due to the child’s cancer [21,22,23].

Childhood cancer can also deeply affect parents’ quality of life [24]. In fact, the quality of life of parents of children with cancer is one of the variables most greatly affected during the process of childhood cancer [25]. However, these situations do not affect all families in the same way, and there are some variables that can have an impact on how parents cope with their child’s cancer [26,27]. Social support is one of the most important variables contributing to reduce psychological effects in parents in the long term [28]. Psycho-Oncology is placing special interest in the field of social support [29,30] because it provides key information on how patients and their relatives face and cope with cancer. Social support has, in fact, proved to reduce stress levels in patients [6].

Social support has been widely studied as a factor that can improve individuals’ coping mechanisms when facing potentially stressful situations [25] and their well-being [27]. Social support plays a positive role in the quality of life of parents of children with cancer [16]. Positive and significant relations have also been observed between perceived social support and mental health in mothers of children with leukemia, who showed lower psychological symptoms and improved satisfaction with life [31].

Support is an exchange of help provided by one person to another, involving a set of social resources that individuals perceive as available at a given time [32]. Support relates to feeling valued and assisted within a social network of mutual support [33]. It is also defined as an interpersonal transaction of help that occurs between a source of support (partner, family, friends, or community) and a recipient of help. This transaction implies emotional support, material help, and information in a specific context [16]. By considering the different sources of support and the different types of support parents can provide (emotional, instrumental, and informational), we are considering the ecological context of parents of children with cancer as suggested by ecological models [3,34]. Additionally, social support is more effective and beneficial the more specific it is regarding the purpose for which is being provided [34,35]. Individuals can receive support, but also provide it. When social support is provided, it can have a positive impact on individuals’ coping mechanisms when facing stressful situations [36]. A balanced exchange of support improves individuals’ well-being [37]. Conversely, imbalances, such as feeling overloaded due to providing more support than the one received or feeling “in debt” due to receiving more support than providing it, can have a negative impact [38].

There are not many studies analyzing social support in its double dimension, that is, the dimension of satisfaction with the support received and satisfaction with the support provided to others. A person can provide support and receive it at the same time, and it is important to know the impact of this double function of support on variables, such as stress and life satisfaction in parents of children with cancer [6,36]. Cancer has a considerable impact on parents’ satisfaction with life, being one of the main variables affected during the process childhood cancer [39]. The different situations and challenges related to the child’s treatments and the disruption of family habits are sources of stress which can often lead to low satisfaction with life [40]. The positive effect of social support can play an essential role in parents’ satisfaction with life [41,42].

**Figure 1:**
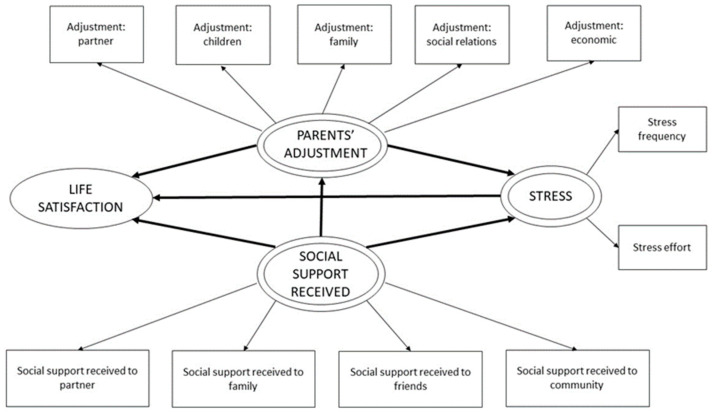
Conceptual model of social support received.

**Figure 2:**
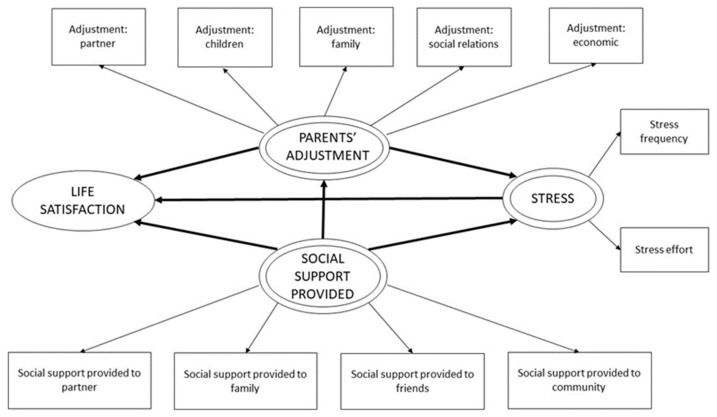
Conceptual model of social support provided.

Previous studies have analyzed some of the relations between the variables described, proving that social support received helps reduce parents’ stress [43,44]. It has also been observed that both social supports received and provided by parents to their network increases their life satisfaction [43,44]. We also know that parents’ adjustment relates negatively to social support received and provided, meaning that providing and receiving support reduces the impact of the disruptions they experience [45]. Considering the above-mentioned findings, the purpose of the present study is to build a model including these variables and, with it, improve and deepen our understanding on how they relate in parents of children with cancer. The potential practical implications derived from this model are significant. The objective is to suggest an integral exploratory model (Figure 1 and Figure 2) to analyze the effect of social support received and provided by parents, parents’ adjustment, and stress on their satisfaction with life. We also aim to examine how these variables relate to each other based on a structural equation model estimated by exploratory least squares (PLS-SEM).

Despite the information we already know on the relations between these variables, the model proposed is exploratory; these relations have never been described jointly and there are not any previous models of structural equations on which to base our analyses. Therefore, the present study and the model included in it suggest the following hypotheses based on results obtained from multiple regression analyses in previous studies:

Social support received:

**H1:**  *Satisfaction with social support received has a direct negative and significant effect on stress.*

**H2:**  *Satisfaction with social support received has a direct negative and significant effect on parents’ adjustment.*

**H3:**  *Satisfaction with social support received has a direct positive and significant effect on satisfaction with life.*

**H4:**  *Perceived stress has a direct negative and significant relation with satisfaction with life.*

**H5:**  *Parents’ adjustment has a direct positive and significant relation with stress.*

**H6:**  *Parents’ adjustment has a direct negative and significant relation with satisfaction with life.*

Social support provided:

**H7:**  *Satisfaction with social support provided has a direct negative and significant effect on stress.*

**H8:**  *Satisfaction with social support provided has a direct negative and significant effect on parents’ adjustment.*

**H9:**  *Satisfaction with social support provided has a direct positive and significant effect on satisfaction with life.*

**H10:**  *Perceived stress has a direct negative and significant effect on satisfaction with life.*

**H11:**  *Parents’ adjustment has a direct positive and significant effect on stress.*

**H12:**  *Parents’ adjustment has a direct negative and significant effect on satisfaction with life.*

## 2. Materials and Methods

### 2.1. Participants

A total of 112 parents of children with cancer participated in the study. Children received treatment at the Mother and Child Hospital in Málaga (Spain). All parents participated in the study voluntarily. Inclusion criteria for the sample were the following: parents or tutors of patients aged between 0 and 21 years with cancer diagnosis. Exclusion criteria were the following: other relatives of patients who were not parents or legal tutors or whose child had passed away. Sociodemographic data are presented in Table 1.

### 2.2. Procedure

Participants received informed consent about the procedure that would be conducted. The study was approved by the Ethical Committee on Scientific Research of the Regional Government of Andalusia (Spain), CEI 2017. Upon signing the informed consent form, participants completed the questionnaire designed to assess stress, adjustment of parents, social support received, and provided and life satisfaction.

Data were analyzed through the structural equation model based on the estimated variance by partial least squares (PLS-SEM) to model simultaneous relations between multiple constructs [46]. This model explains the variance of the endogenous construct (life satisfaction) through different latent predictive variables (adjustment of parents, perceived stress, and social support received and provided). Amongst the reasons for using the PLS-SEM model is the use of formative constructs and the interest in predicting [47]. In our case, the descriptive analysis of items that compose the measurement scale confirmed the no-normality of data based on asymmetry coefficients, since they did not meet the recommended threshold of ±1 [48]. This justifies the use of PLS-SEM model. Statistical software Smart PLS (version 3.3.2) was used for data analysis [49].

### 2.3. Measures

*Questionnaire for the assessment of adjustment of parents of children with cancer*

Designed ad hoc by Hombrados-Mendieta and Martos-Méndez [44,45], this questionnaire measures situations that take place in different areas of parents’ lives over the course of the child’s cancer. It asks participants about the changes or disruptions brought about in different areas of their lives due to the child’s cancer: partner (items 1 to 8), children (items 9 to 16), extended family (17 to 22), social relations (23 to 27), and economic and employment situations (items 28 to 30). The total number of items in this questionnaire is 30. There are 5 answer options for each item to assess the level of agreement or disagreement (“1” means fully disagree and “5” means fully agree). Scores from 7 items were rotated to obtain negative data on parents’ adjustment to the situations described in each item (2, 3, 6, 9, 12, 17, and 23). This way, higher scores in these items suggested lower adjustment of parents to the child’s cancer. Cronbach’s Alpha for the full scale is α = 0.79. The following indexes were obtained in each dimension of adjustment: partner α = 0.69, children α = 0.70, family α = 0.50, social relations α = 0.54, and economic and employment situation α = 0.64. Although Cronbach’s Alpha is slightly low in some dimensions, the general Alpha of the instrument is within the allowed standards. Nunnally and Bernstein [50] note that moderate reliability (for instance, a value of 0.7) can be considered a satisfactory Cronbach’s Alpha value during the initial stages of validation or predictive research. Hair et al. [48] also note that Alpha coefficients ranging from 0.60 to 0.70 are considered acceptable in exploratory research, while values from 0.70 to 0.90 are considered satisfactory.

*Frequency and Satisfaction with Social Support Questionnaire (QFSSS)*

The Frequency and Satisfaction with Social Support Questionnaire (QFSSS) [51] was used to measure parents’ satisfaction with the social support they received and provided, and, more specifically, the type of support (emotional, instrumental, and informational) received and provided to each source of their social network (partner, family, friends, and members of the community). The questionnaire comprises 12 items about support received, and 12 items about support provided. There are five answering options, ranging from “1” meaning “completely unsatisfied” to “5” meaning “very satisfied”. Participants were asked about their degree of satisfaction with the social support received and provided by them (sources and types). In this study, an average score on social support received and provided measured based on the three types, and the four sources was used.

Cronbach’s Alpha for the full scale is α = 0.96. The following are some examples of items included in the section of satisfaction with social support received: you feel loved and cared for, and liestened to when you need to talk and express emotions (emotional support from partner); your partner does things for you, such as helping with daily house chores or the care of the child (functional support). The following are examples of some items included in the section of satisfaction with social support provided: you give them useful advice or information to solve their questions, problems or daily tasks (informational support provided to friends); you are happy to do specific things for them, such as helping them with daily house chores or the care of their child (informational support provided to the community).

*Pediatric Inventory for Parents (PIP)*

The Spanish version of the Pediatric Inventory for Parents by Streisand et al. [52], adapted and validated by Del Rincón et al. [53], was used. This questionnaire assesses the stress caused by the daily different situations faced by parents of children with cancer. It comprises one scale of frequency and another of effort. Each scale included 42 questions related to situations parents must face during their child’s cancer. Participants must first answer about the frequency of each item by choosing from “1” meaning “never” to “5” meaning “very often”, and about the effort that such activity involves for them; choosing from “1” meaning “none” to “5” meaning “very much”. The following are two examples of items included in the frequency scale and the effort scale, respectively: How often do you find it difficult to sleep?, how difficult is it for you to attend your child’s medical tests and treatments? Cronbach’s Alpha of the full scale is α = 0,95.

*Life Satisfaction Scale*

The Life Satisfaction Scale from Pavot and Diener [54] was used. This scale offers a general index of life satisfaction. Life satisfaction is understood in this scale as a general construct of subjective well-being. It is a multidimensional scale composed of 5 items answered through a Likert-type scale of 7 points (1 = completely disagree and 7 = completely agree). The following is an example of an item included in this scale: my life is very close to how I would like it to be in most cases. Cronbach’s alpha of the full scale is α = 0.87.

## 3. Results

### 3.1. Descriptive Characteristics of Findings

As it can be seen in Table 2, descriptive analyses were conducted to measure participants’ stress levels, their adjustment in each dimension, social support received and provided, and their satisfaction with life. Results show that parents express medium-high stress levels when facing situations related to their child’s cancer. In what concerns parents’ adjustment, results show they have medium-high difficulties to apply the necessary adaptations to the situation. Parents also reported receiving medium levels of support and providing medium support levels. Finally, they expressed medium-low levels of satisfaction with life.

### 3.2. Theoretical Model

The theoretical model suggested is based on research of the literature on the latent variables (composites) considered. Although it is a confirmatory analysis, the two proposed models are exploratory models based on the empirical relationships researched in the literature in the introduction. Figure 1 and Figure 2 show the two models.

### 3.3. PLS Analyses

Data were analyzed using a PLS-SEM model, which allows to work with complex models with several latent variables, indicators or observed variables, and with relations between latent variables and indicators [48,55].

The final sample was composed by 112 participants. GPower 3.1.9.2 software, a statistical power analysis program usually applied in social and behavioral research [56], was used to determine the size of the sample. With a statistical power of 1 − β = 0.80, an effect size of 0.17 and a significance level of 0.05—the minimum sample required is 106.

The disjoint two-step approach was applied to estimate the second-order model [55,57]. During the first stage, scores from latent variables in the measurement models were estimated after removing second-order constructs, and transferring the relation between second-order constructs and the corresponding first-order constructs. Measurement models were also assessed during this first stage. In a second stage, latent scores from estimated first-order constructs were included as second-order indicators, thus making them first-order constructs. During the second stage, the structural model was only assessed as if it were a first-order model [56,58,59] with a previous analysis to detect any severe collinearity issues. The assessment was conducted through a 500sample bootstrapping, contrasted with two tails, with the percentile bootstrap to retain indicators, VLs and those paths that reached a significance level of 0.05. For the assessment of the model’s predictiveness, R^2^ values of endogenous variables were analyzed. The size of the effect was also obtained (q^2^), which provides information on the increase of R^2^ from endogenous variables when the corresponding precedent LV predictor is omitted.

### 3.4. The Measurement Model

The models are a compound models estimated in Mode A, meaning that indicators relate reflectively with the construct. The measurement models meet reliability results. According to composite reliability, measurements are robust in terms of internal consistency and reliability [50,60]. Composite Reliability (CR) of measurements varied between 0.81 and 0.89, which is above the recommended threshold of 0.70 [61]. Additionally, Average Variance Extracted (AVE) exceeded 0.50 for each measurement, in line with recommendations [55]. Square roots of AVEs were higher than crossed correlations between latent variables for all cases, thus supporting that LVs measure different concepts [48]. HTMT ratio (HeteroTrait-MonoTrait) did not exceed the established threshold of 0.90 [62]. Furthermore, discriminant validity was confirmed by extracting factor loadings and cross loadings. All items had a loading for its corresponding construct from a minimum of 0.72 to a maximum of 0.97, and higher for their corresponding construct than for any other [63]. This result also supports the validity of these indicators as representative of different LVs.

### 3.5. The Structural Model

**Figure 3:**
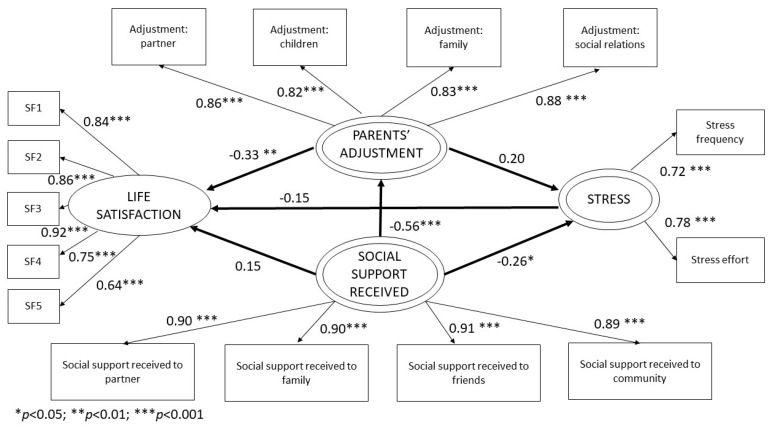
Structural social support received model results.

**Figure 4:**
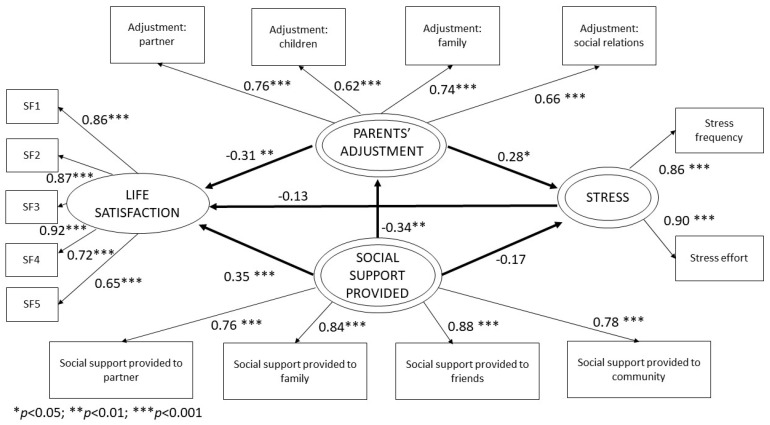
Structural social support provided model results.

Figure 3 and Figure 4 shows the estimated structural model. In the model of social support received we can observe that all path coefficients are statistically significant (*p* < 0.05), except Received Social Support on Life Satisfaction, Stress on Life Satisfaction, and Adjustment of Parents on Stress. The R^2^ coefficient value for Adjustment of Parents was 0.32, 0.16 for Stress, and 0.26 for Life Satisfaction, thus showing that the model has a moderated predictive effect for the Stress, and substantial for the Adjustment of Parent and Life Satisfaction [64]. The influence of satisfaction with the Social Support Received with respect to Adjustment of Parents is negative; the more Social Support Received, the less negative adjustment experienced by parents of children with cancer, a result that is consistent with the expected sign (β = −0.56). On the other hand, the Social Support Received has a significant and negative influence on Stress, in such a way that the more social support received, the less stress parents experience (β = −0.26). As far as Life Satisfaction is concerned, only the direct effects of Adjustment of Parents are significant with a negative effect of (β = −0.33). That is, the greater the adjustment experienced by parents, the lower their life satisfaction. However, the relation between Social Support Received and Life Satisfaction (β = 0.15), the relation between Stress and Life Satisfaction (β = −0.15), and the relation between Adjustment of Parents and Stress (β = 0.20), were not significant. The size of the effect of each LV was calculated through f^2^ values, most effects are weak tending to moderate, except for the Social Support Received on Adjustment of Parents which is strong (Table 3). The model does not show any severe collinearity issues between variables; VIF values (Variance Inflation Factor) did not exceed 5 [55,65] or 3.3 as considered by other authors [66]. The predictiveness of the model was also analyzed by calculating effect size rates (q^2^). Results exceeded the recommended threshold (q^2^ > 0), thus confirming the appropriate predictive validity of the model [67].

In the model of social support provided, all path coefficients are statistically significant (*p* < 0.05), except for Social Support Provided on Stress and Stress on Life Satisfaction. The R^2^ coefficient value for Adjustment of Parents was 0.12, 0.14 for Stress, and 0.36 for Life Satisfaction, thus showing that the model has a moderated predictive effect for the first two and substantial for the last one [64]. The effect of Social Support Provided compared to Adjustment of Parents is negative, meaning that when Support Provided is higher the adjustments that parents have to make in their lives are less negative (β = −0.34). Social Support Provided showed a significant and positive effect over parents’ Life Satisfaction; the higher the Support Provided the higher the Life Satisfaction, with a beta coefficient of β = 0.35. Adjustment of Parents experienced showed a significant and positive relation with perceived Stress (β = 0.28); the higher the changes experienced, the higher the stress levels. Adjustment of Parents also affect parents’ Life Satisfaction, with a significant and negative effect (β = −0.31); the higher the changes experienced, the lower parents’ life satisfaction. However, the relation between Social Support Provided and Stress (β = −0.17), and the relation between Stress and Life Satisfaction (β = −0.13), were not significant. The size of the effect of each LV was calculated through f^2^ values and ranged from weak to moderated (Table 4). The model does not show any severe collinearity issues between variables; VIF values (Variance Inflation Factor) did not exceed 5 [55,65] nor 3.3 as considered by other authors [66]. The predictiveness of the model was also analyzed by calculating effect size rates (q^2^), highlighting the effect size of Social Support Provided on Life Satisfaction. Results exceeded the recommended threshold (q^2^ > 0), thus confirming the appropriate predictive validity of the model [67].

## 4. Discussion

The estimated models highlight the importance of better understanding how the two dimensions of social support impact parental adjustment, stress, and life satisfaction of parents of children with cancer. Most of the research on social support places its main focus on analyzing social support received, with few studies examining the influence that social support provided may have on parents’ quality of life. Previous studies have shown that the social support received is a source of resources helping parents cope with the difficult situation of childhood cancer [68]. However, the social support that parents provide to their own network is also a fundamental variable in the management of this process, as the results of this study show.

With respect to the hypotheses of the study, and to fulfill the objective of analyzing social support in its double dimension (support received and support provided), these have been divided into two models. A model of the social support received from parents of children with cancer, and another model of the social support provided by parents of children with cancer have been proposed. The results of each model are discussed below.

### 4.1. Social Support Received Model

Three hypotheses from the initial six suggested were confirmed. Hypothesis 1 is confirmed by the direct negative and significant effect of social support received seen on stress. Social support received by parents predicts a reduction in stress. These results confirm the importance in these situations of receiving support to cope with daily tasks, and to cope more effectively with potentially stressful situations derived from cancer [43]. This is highly relevant when providing support to these families, as one of the strategies that can best help them manage the stress of the situation, they are experiencing is the social support received from the network [24] (partner, family, friends, and community/associations). Regarding Hypothesis 2, satisfaction with social support received proved to have a direct negative and significant effect on parents’ adjustment. Social support received also predicts less adjustment in parents’ lives. This is very relevant as parents experience many novel and difficult situations that involve negative adjustments related to various aspects (adjustments in the couple, in the relationship with children and other family members, in social life). The social support that parents receive from the various sources of support helps them to reduce these negative adjustments. Hypothesis 3 was also confirmed by the direct positive and significant effect observed between social support received and satisfaction with life. The social support received would not be directly and significantly related to parents’ life satisfaction. However, in previous studies, we found that the emotional social support received from the partner has a positive impact on life satisfaction, with no other social support received being significant in increasing life satisfaction [69]. Hypothesis 4 predicted a direct negative and significant effect of perceived stress on satisfaction with life. Yet, an increase in stress does not seem to predict a decrease in parental life satisfaction. Hypothesis 5 suggested a direct positive and significant effect of parents’ adjustment on stress; however, parental adjustment did not relate to perceived stress. Finally, Hypothesis 6 suggested a direct negative and significant effect of parental adjustment on satisfaction with life. We see a relation between parental adjustments and life satisfaction, where higher adjustments made by parents relate to lower satisfaction with life.

### 4.2. Social Support Provided Model

Few studies analyze the effect of providing social support on parents’ quality of life. Social support provided by parents to their network can be a significant variable for the improvement of how they handle the child’s cancer. The suggested model between latent variables related to social support provided by parents has shown results that ought to be considered. Some of the variables included in this model have been widely studied in the past, such as life satisfaction and stress in parents of children with cancer. However, these variables had not been studied before in relation with adjustment of parents and the social support they provide to their network. Results showed significant relations between the latent variables included in the model. Out of the six hypotheses suggested for this model, four were confirmed. Hypothesis 7 suggested a direct negative and significant effect of satisfaction with social support provided on stress. However, the relation between social support provided and perceived stress was not statistically significant. Hypothesis 8 suggested a direct negative and significant effect of satisfaction with social support provided on parental adjustment. Social support provided was also found to predict a decrease in adjustment of experienced by parents. The range of adjustment experienced by parents during the process of childhood cancer can be highly complex, and involve great difficulties, which increases parents’ perception of stress [2,18]. Hypothesis 9, which suggested a direct positive and significant effect of satisfaction with social support provided on satisfaction with life, was confirmed: social support provided by parents relates to satisfaction with life significantly and positively. The variable of Life Satisfaction is closely linked to personal fulfilment [70,71], and it seems to have a significant relation with the ability to provide support to others and serve as an example in complex circumstances, such as childhood cancer. This might be because parents, on many occasions, are the ones who become an example for other members of the family who are going through similar situations, and even for other parents of children with cancer through support groups, associations, etc. [4]. This can help parents feel in control over their lives and become active subjects in developing resources, coping with difficult situations and providing support [20]. This phenomenon can have a direct effect on parents’ life satisfaction, who are able to help others and therefore feel useful and make the most of their experience by helping themselves and others. Hypothesis 10 suggested a direct negative and significant relation between perceived stress and satisfaction with life; however, this relation was not statistically significant. Hypothesis 11 predicted a direct positive and significant relation between parental adjustment and stress, which was confirmed by the model, where parents’ adjustment showed a positive and significant relation with perceived stress. Finally, hypothesis 12 predicted a direct negative and significant relation between parents’ adjustment and their satisfaction with life: results show that higher disruptions experienced by parents relate to lower levels of life satisfaction; greater changes experienced by parents relate to a higher negative effect on their life satisfaction. It is worth noting that economic and labor changes did not prove significant in this model. However, there are other studies that confirm that families of children with cancer experience economic and labor changes [21,34,72].

Results highlight the importance of analyzing support not only from the perspective of receiving it, but also providing it, since providing support has proved to be beneficial satisfaction of life of parents.

With the analysis of the two models of social support received and social support provided, we have verified the importance of the variables studied in the parents’ situation. Knowing which types of support are more efficient for specific issues is highly relevant [73].

## 5. Conclusions

Results obtained from the two models highlight the importance of the analysis of the social support in terms of the exchange that takes place between parents and their support networks [38], since the fact that parents, and obtaining support, are able to provide support to their network, has great benefits for their quality of life and well-being. Social support does not occur in a unidirectional manner, but rather a bidirectional transaction that takes place, and it is necessary to analyze the benefits of such an exchange of support. On the other hand, knowing what kind of support is most effective for what specific issue is important [34]. In the results of the study, we see that the social support received is shown to be effective in reducing stress, but it is the social support provided that predicts an improvement in life satisfaction. It is also interesting to note that both, social support received and social support provided contribute to reducing the adjustment of parents. These findings are of special relevance to improve the quality of the support provided to families of children with cancer, adapted to their real needs. In the current era of precision medicine which aims at providing specialized treatment based on the specific needs of individuals [74], it is a priority to know and understand the reality of cancer patients and their families [75]. Many factors that affect them go beyond biomedical factors, and these can impact the treatment process and the care provided to them. Cancer is a serious illness that affects a great number of people, and which will keep having a strong impact on our society. Within the term ‘cancer’ there is a broad variety of realities and a great number of different types and patients with very different characteristics [76]. In particular, childhood and adolescence cancer have very specific features related to the family sphere, which must be considered when designing treatment and care. It is essential to provide scientific knowledge on the psychosocial factors surrounding childhood cancer. This will allow us to improve the medical care provided in the current times of precision medicine [77].

### 5.1. Practical implications

It would be desirable to develop guidelines for parents to provide the support they need, as this support has implications for stress reduction and less negative adjustment. Normally, people draw their social support from their natural support system, which consists mainly of the social networks of family and friends. This natural network provides a wide range of types of support. However, as [78] points out, sometimes the network is willing to provide support, but does not know how to provide it. Often, it is necessary to work with family members or the closest support network of the person experiencing the problematic situation. This is especially true for people suffering from cancer or other serious illnesses, as the illness may disrupt the support network and feelings of fear or feelings of avoidance may arise among the members of the network. Not knowing the appropriate way to deal with parents of children with cancer can lead to anxiety about the interaction. Therefore, this can produce undesirable and counterproductive effects to the perception of support that may lead to physical avoidance of the patient or avoidance of communication about the situation. Intervention is needed to assess the needs of parents and to develop competencies in the provision of help from their immediate support network. On the other hand, the social support provided is effective in increasing parental life satisfaction. It is important for parents to be active and to help other parents, or to participate in associations. This may lead to an active coping with the child’s cancer and, in this sense, intervention guidelines should be developed to reduce the feeling of helplessness caused by the child’s cancer through the involvement and empowerment of this group. Understanding the role of social support received and social support provided on stress, parental adjustment and life satisfaction may have important practical implications for the design of interventions to improve their quality of life. It seems clear that improving the quality of life of children with cancer and their families requires the treatment of psychosocial aspects [79] and, for this, it is necessary to know how these variables are related in people who are suffering from this situation [80]. These practical implications can be made available to professionals who provide care to families, such as nurses [74,76,77], so they can give families personalized care by knowing the key role played by support networks and the nature and positive impact of such support.

### 5.2. Limitations and Strengths of the Study

The fact that all participants are from Málaga (Spain) and nearby areas limits the generalization of results to other contexts and cultures. It would be of interest to replicate the model in other geographical contexts. The size of the sample is also a limitation, since it ought to be larger to detect the size of weak effects.

Regarding the characteristics of the sample, there were more female than male participants, so it would be important to balance the gender of participants in the study to analyze support in a differential manner. However, it is also important to bear in mind that this reflects a social reality, where the number of women responsible for the care of sick children is significantly higher compared to men. Additionally, women tend to be more willing to participate in this type of research [81]. In this sense, it would also be interesting in future research to study gender and age differences, since the impact of these variables can be significant.

In future studies, variables could also be analyzed according to the different stages of the childhood cancer and the different types of cancer. It would also be necessary to study how the different types of diagnoses and hospitalization periods impact parents. It would be of interest to suggest longitudinal designs that would allow to discover other adaptation and coping aspects related to childhood cancer from parents’ perspective. Future research could suggest explicative models that could include variables relevant to other factors, such as parents’ health, family satisfaction, or resilience. Future research could also explore and study parents’ adjustment through different instruments of higher statistical solvency, including additional analytical approaches that could provide further information on the phenomenon analyzed in the present study.

One of the main strengths of the present study is that it provides new insights on the relations between the variables related to facing childhood cancer by parents and the effect that cancer has on parents’ quality of life. Likewise, broadening the knowledge on these relations is of great value to improve parents’ adaptation to cancer, clinical practice, and acting protocols. There are few studies that focus on estimating models with psychosocial variables related exclusively to parents and in the context of childhood psycho-oncology. Finally, it is also relevant to consider the practical implications of the results obtained from the present study, and to apply them to the daily activities of professionals who provide psychosocial care and support.

## Figures and Tables

**Table 1:** Demographic and clinical data (*n* = 112).

Measures	Mean (Standard Deviation)	Percentage % (N)
Age (father/mother/tutor)	41 (6.93)	
Age (child with cancer)	8 (5.02)	
Gender (parent/tutor)		
Male		33.9 (38)
Female		66.1 (74)
Marital Status		
Single		3.6 (4)
Married		80.4 (90)
Divorced		3.6 (4)
Separated		3.6 (4)
Widow		0.9 (1)
Unmarried partner		1.8 (2)
Lives with partner		6.3 (7)
Gender (child with cancer)		
Male		58 (65)
Female		42 (47)
Type of cancer		
Leukemia		53.6 (60)
Ewing sarcoma		8.9 (10)
Lymphoma		8 (9)
Medulloblastoma		4.5 (5)
Neuroblastoma		4.5 (5)
Rhabdomyosarcoma		2.7 (3)
Hepatoblastoma		2.7 (3)
Astrocytoma		1.8 (2)
Other		13.4 (15)

**Table 2:** Descriptive statistics of study variables (*N* = 112).

Variable	Mean	Standard Deviation
Social support received to partner	2.87	0.94
Social support received to family	2.77	0.83
Social support received to friends	2.60	0.82
Social support received to community	2.59	0.84
Emotional social support received	2.93	0.66
Instrumental social support received	2.83	0.73
Informational social support received	2.74	0.74
Social support provided to partner	3.05	0.79
Social support provided to family	2.76	0.84
Social support provided to friends	2.68	0.81
Social support provided to community	2.57	0.85
Emotional social support provided	2.94	0.66
Instrumental social support provided	2.82	0.73
Informational social support provided	2.74	0.75
Parents’ adjustment: total	2.65	0.50
Parents’ adjustment: partner	2.33	0.85
Parents’ adjustment: children	2.72	0.68
Parents’ adjustment: family	2.73	0.68
Parents’ adjustment: social relations	2.68	0.66
Parents’ adjustment: economic	2.77	1.11
Stress frequency	3.26	0.66
Stress effort	2.76	0.82
Life satisfaction	3.53	1.17

**Table 3:** Path coefficient, effect size, magnitude, and significance of the structural social support received model paths.

Paths	β	f^2^	Type f^2^	T Stat.	*p*-Value
Social support received on Stress	−0.26	0.05	Weak	1.99	0.023 *
Social support received on Parents’ adjustment	−0.56	0.46	Strong	9.01	0.000 ***
Social support received on Life satisfaction	0.15	0.02	Weak	1.23	0.110
Stress on Life satisfaction	−0.15	0.02	Weak	1.42	0.077
Parents’ adjustment on Stress	0.20	0.01	Weak	1.47	0.071
Parents’ adjustment on Life satisfaction	−0.33	0.10	Weak	2.77	0.003 **

Note. T Stat. *t*-student statistic. *, **, ***: significance level of 5%, 1%, and 0.01%, respectively.

**Table 4:** Path coefficient, effect size, magnitude, and significance of the structural social support provided model.

Paths	β	f^2^	Type f^2^	T Stat.	*p*-Value
Social support provided on Stress	−0.17	0.03	Weak	1.50	0.133
Social support provided on Parents’ adjustment	−0.34	0.13	Moderate	3.35	0.001 **
Social support provided on Life satisfaction	0.35	0.17	Moderate	3.91	0.000 ***
Stress on Life satisfaction	−0.13	0.02	Weak	1.35	0.177
Parents’ adjustment on Stress	0.28	0.08	Weak	2.18	0.030 *
Parents’ adjustment on Life satisfaction	−0.31	0.12	Weak	2.92	0.003 **

Nota. T Stat. *t*-student statistic. *, **, ***: significance level of 5%, 1%, and 0.01%, respectively.

## Data Availability

The data presented in this study are available on request from the corresponding author. The data are not publicly available due to ethical and privacy issues of participants.
